# 

**Published:** 1913-12-27

**Authors:** 


### Appointments.

Mr. W. C. Rivers, M.R.C.S., L.R.C.P., resident medical officer at Barrasford Sanatorium, Newcastle-on-Tyne, has been appointed tuberculosis officer to the West Riding. A Charing Cross Hospital man, he held appointments at Mount Vernon Hospital and the Throat Hospital, Golden Square. He has been in charge at Barrasford for five years.

Col. J. J. Pratt, I.M.S., F.R.C.S., has been appointed surgeon to the Seamen's Hospital Society (Dreadnought) with duties at the Albert Dock Hospital, to which the London School of Tropical Medicine is attached. Col. Pratt has had a distinguished career in India, and was Herbert prizeman and Montefiore medallist on leaving Netley. On retirement he vacated Lucknow, which is the premier charge in the United Provinces with a large civil hospital.

### Gifts and Bequests.

Among the legacies for Brompton Hospital announced since the last Court are £5,000 from the late Sir William Dunn, Bart.; £1,000, duty free, from the late Miss Alice Sarah Price; and £1,000 from the late Miss Hannah Elizabeth Gartside. The committee, while acknowledging a donation of £50 to the Sanatorium Chapel Fund from Mr. E. S. Barker, point out that work on the chapel has been suspended from want of funds. A further £1,000 is required for this purpose. The number of applications for admission, as well as of out-patients, continued to decrease.

Mr. Ephraim Yeo, of Ramsgate, who died on August 24, left stock of the nominal value of £200 to his housekeeper for life, with remainder to Newton Hospital, Newton Abbot; stock of the nominal value of £1,100 to his servant Sarah Ailes for life, with remainder to Torbay Hospital and Dispensary; stock of the nominal value of £480 to his servant Sarah Ailes for life, with remainder to Newton Hospital; and stock of the nominal value of £462 10s. to Louisa Barnes for life, with remainder to Newton Hospital.

The Hon. Treasurer of the Clapham Maternity Hospital (under medical women) acknowledges the following donations to the buildings fund of the hospital now in process of reconstruction: Corporation of London, £25; the Worshipful Company of Vintners, £10 10s.

Sir William Tate and Mr. Edwin Tate have given £1,600 and £900 respectively toward building a nurses' home at the West Ham and Eastern General Hospital.

Mr. Austen Chamberlain has received £200 from the Government of Trinidad and £150 from the Government of Sierra Leone for the extension and development of the London School of Tropical Medicine.

Miss Margaret Emily Gaskell, of Manchester, daughter of Mrs. Gaskell, the novelist, who left estate valued at £50,223 gross, has bequeathed £4,500 to the Memorial Nursing Home, Manchester, £1,500 to the Ardwick District Nurses' Home, and £1,000 to the Ancoats Hospital.

At a meeting of the working committee of the Ladies' Association of the Royal Waterloo Hospital for Children and Women recently the Duchess of Albany, who presided, presented the treasurer, Mr. J. Topham Richardson, with a cheque for £500 from an anonymous donor.

The After-Care Association for Poor Persons Discharged Recovered from Asylums for the Insane, Church House, Dean's Yard, Westminster, has received £15 15s. from the Drapers' Company.

The London Hospital has received £100 from Sir John Glover in response to Mr. Holland's appeal.

A cot will be named at the West End Hospital for Diseases of the Nervous System in respect of a donation of £300 from Miss Goredy. A contribution of £50 has also been received from the Worshipful Company of Grocers.

Mr. Frederic William Goodliffe, of Bournemouth, has, subject to some small annuities and bequests, left the residue of his property to accumulate during the life of his wife, and on her death he left £1,000 to the Royal Victoria and West Hants Hospital; £4,000 among nine other institutions, and the ultimate residue of his property "for the benefit of persons or institutions which I shall designate by a list to be hereinafter signed by me." There would appear not to have been such list prepared, as it is not filed with the will.

### A Special Offer.

Officials responsible for the administration of institutions will find The Hospital of the greatest practical service when a vacancy occurs upon their staffs, since an announcement in its columns secures the widest publicity among those especially trained to Institutional Work. Through the agency of this Journal it is possible to obtain the most efficient officers in every branch of Institutional Life. In these circumstances particular attention is directed to the fact that Special Rates are allowed to those Institutions that agree to send all their public notices for insertion in this section of The Hospital during 1914. Full particulars can be obtained on application to The Manager, The Hospital, The Hospital Building, 28 and 29 Southampton Street, Strand, London, W.C.

### Manager's Notices.

**Letters relating to the publication, sale, and advertising departments of "The Hospital" must be addressed to The Manager, "The Hospital," The Hospital Building, 28 and 29 Southampton Street, London, W.C.**

**Advertisements:**

To ensure insertion, all advertisements for The Hospital must reach the Manager not later than Wednesday morning in each week. For scale of charges see pages v and vii.

**Special Rates** will be quoted for those institutions that agree to send all their vacancy, contract, and public notices to The Hospital each year, so as to make it a file of such announcements for ready reference.

**Subscriptions** may begin at any time, and are payable in advance. Cheques and Post-Office Orders should be crossed London County and Westminster Bank, Covent Garden Branch, and made payable to the Manager of The Hospital.

Rates of Subscription (payable in advance).

| *United Kingdom.* | | *Foreign and Colonial.* | |
|---|---|---|---|
| Three Months ... | ... 2s. 0d. | Three Months ... | ... 2s. 6d. |
| Six Months ... | ... 4s. 0d. | Six Months ... | ... 5s. 0d. |
| Twelve Months ... | ... 6s. 6d. | Twelve Months ... | ... 8s. 8d. |

*SUBSCRIPTION FORM.*

*Please send me* The Hospital *for..............months, commencing with your issue of.............................., for which please find enclosed............................*

*Name*.....................................................

*Address* ...................................................

.................................................

*Date*...................... ...............

*To* ....................................... *Newsagent.*

**Remittances:**

**It is especially requested that remittances be made by Cheque or Postal Order, and in halfpenny stamps only when the amount is under sixpence.**

Rates of Subscription: Twelvemonths, 6 6; Six months, 4/-; Three months, 2/-, post free to any address in the United Kingdom. Abroad, Twelvemonths, 8/8; Six months, 5/-; Three months, 2/6, post free.—Address The Subscription Dept., "The Hospital," The Hospital Building, 28/29 Southampton Street, Strand, London, W.C.

**IMPORTANT NOTICE TO INSTITUTIONAL AUTHORITIES.**

**We are desirous of directing the attention of our readers to the new**

### BOOK SALOON

**which has recently been inaugurated at**

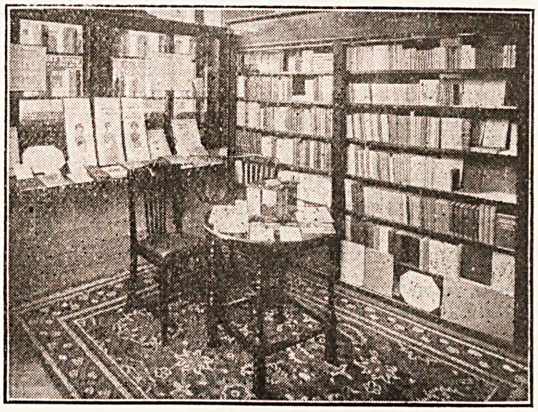

A CORNER OF THE BOOK SALOON.

**"THE HOSPITAL" Building, 28 & 29 Southampton Street,**
**STRAND, LONDON, W.C.**

